# Differential Reshaping of Skin and Intestinal Microbiota by Stocking Density and Oxygen Availability in Farmed Gilthead Sea Bream (*Sparus aurata*): A Behavioral and Network-Based Integrative Approach

**DOI:** 10.3390/microorganisms12071360

**Published:** 2024-07-02

**Authors:** Socorro Toxqui-Rodríguez, Paul George Holhorea, Fernando Naya-Català, Josep Àlvar Calduch-Giner, Ariadna Sitjà-Bobadilla, Carla Piazzon, Jaume Pérez-Sánchez

**Affiliations:** 1 Nutrigenomics and Fish Growth Endocrinology Group, Institute of Aquaculture Torre de la Sal (IATS, CSIC), 12595 Castellón, Spain; socorro.toxqui@csic.es (S.T.-R.); paul.holhorea@csic.es (P.G.H.); fernando.naya@iats.csic.es (F.N.-C.); j.calduch@csic.es (J.À.C.-G.); 2 Fish Pathology Group, Institute of Aquaculture Torre de la Sal (IATS, CSIC), 12595 Castellón, Spain; ariadna.sitja@csic.es (A.S.-B.); carla.piazzon@csic.es (C.P.)

**Keywords:** gilthead sea bream, microbiota, behavior, gene expression, skin, intestine, stocking density, Gh/Igf system

## Abstract

Fish were kept for six weeks at three different initial stocking densities and water O_2_ concentrations (low-LD, 8.5 kg/m^3^ and 95–70% O_2_ saturation; medium-MD, 17 kg/m^3^ and 55–75% O_2_ saturation; high-HD, 25 kg/m^3^ and 60–45% O_2_ saturation), with water temperature increasing from 19 °C to 26–27 °C. The improvement in growth performance with the decrease in stocking density was related to changes in skin and intestinal mucosal microbiomes. Changes in microbiome composition were higher in skin, with an increased abundance of *Alteromonas* and *Massilia* in HD fish. However, these bacteria genera were mutually exclusive, and *Alteromonas* abundance was related to a reactive behavior and systemic growth regulation via the liver Gh/Igf system, while *Massilia* was correlated to a proactive behavior and a growth regulatory transition towards muscle rather than liver. At the intestinal level, microbial abundance showed an opposite trend for two bacteria taxa, rendering in a low abundance of *Reyranella* and a high abundance of *Prauserella* in HD fish. This trend was correlated with up-regulated host gene expression, affecting the immune response, epithelial cell turnover, and abiotic stress response. Most of the observed responses are adaptive in nature, and they would serve to infer new welfare indicators for increased stress resilience.

## 1. Introduction

Global aquaculture production has been increasing at a high rate since 1990 [1]. As a consequence, the intensification of production must deal with inappropriate animal stocking densities and impaired health and growth performance [2,3,4]. Hence, significant efforts have been made to support the expansion of more sustainable aquaculture, combining the criteria of economic profitability with enhanced control and regulation in health and welfare assurance schemes [5,6,7]. In that sense, measurements of circulating cortisol levels are the most widely used stress biomarker approach in farmed fish, though its reliability in an aquatic scenario is limited by its large individual variability and dramatic increases by sampling itself [7,8]. Alternatively, behavioral observations [9,10], the external appearance of fish [11,12], and tissue-specific transcriptional features [13,14,15,16,17] arise as successful integrated biomarker approaches for improving the sustainability of intensive fish farming. Thus, linking the quantification of the stress response with fitness traits has the potential to provide new physiological insights through life history, as earlier reported in birds and reptiles [18,19]. Additionally, accelerated male-female sex reversal is also becoming a cumulative index of impaired welfare in protandric hermaphrodite fish such as gilthead sea bream (*Sparus aurata*) [20].

The social context in which fish are kept also influences behavioral traits in schooling fish such as gilthead sea bream [21,22]. Certainly, escape behavior responses in fish subjected to different restraint tests did not exhibit consistent responses with the changes in social environment [23], though high stocking densities reinforced the social cohesion of the population [24,25], while feeding time acts as a main zeitgeber factor or time-giver, defined as the external cue that entrains the biological clock [26]. There is also now evidence that swimming behavior and performance are genetically regulated in gilthead sea bream [27,28], but we are still far from understanding all the regulatory processes from the cell to the whole organism level. In this regard, it is noteworthy that Holhorea et al. [26] displayed a growth-regulation transition from systemic to local growth-regulatory mechanisms in fish stocked at high densities, which might also support a proactive (i.e., bold and aggressive responses, instead of a reactive (i.e., shy and subordinate responses) behavior, according to Koolhaas et al. [29]. This functional feature was accompanied by the up-regulation of antioxidant enzymes and molecular chaperones in addition to the fine adjustments of lipolytic and lipogenic enzymes, which is considered adaptive in nature to efficiently manage hypoxic environments at high fish stocking densities [14]. However, the link between behavioral and metabolic homeostasis with microbial organisms remains to be established, though it is well-known that resident gut microbiota modulates host behavior in humans and terrestrial livestock [30,31,32]. Much of this earlier work regarding bidirectional gut-brain communication has concentrated on digestive function and satiety, but recent research focused on cognitive and psychological effects highlighted the association of changes in cognitive function and gut microbiota composition following acute and chronic stress events. Less is known in fish but given the critical role of microbiota in host function, there is a recognized interest in shifting in this direction in farmed fish [33].

Gilthead sea bream is one of the main cultured fish in the Mediterranean region, and it is well-known that its associated microbial communities play a key role in protection against pathogens, nutrient digestion and absorption, and osmotic regulation [34,35]. In the gut, genetics strongly modulated the mode of action of a given feed additive upon the gilthead sea bream gut microbiota and its interconnection with a wide range of physiological processes at local and systemic levels [36,37]. A shift towards the characterization of microbiota is also being undergone to evaluate and improve fish welfare and nutrition, disclosing clear perturbations of skin [38] and intestinal microbiota composition [39] following episodes of chronic stress. Thus, as stated before, it is clear that the diversity of microbial communities can change the ecosystem and reflect the state of fish under different environmental conditions [40]. Calculating different factors, such as microbial richness, alpha-, and beta-diversity among individuals, emerges as the key to clarifying how aquaculture individuals react as a population [41]. Thus, according to the hologenome theory of evolution, the holobiont (host-microbiome system) might act as a unit of evolutionary selection, facilitating the fast genomic changes of the microbiota and the adaptation of the holobiont to constantly changing environmental conditions [42,43]. Such integration may even account for complex biological phenomena, such as certain behaviors, which have led to the use of the concept “psychobiotics” for the treatment of various neurological and behavioral disorders by targeting the gut microbiota [44,45]. The field is now running to mechanistic studies in humans, but the behavior and microbiota associations are still in an early state in animal production and fish farming in particular. To address some of these pressing knowledge gaps, this study aimed to disclose how behavior and microbiota are related in gilthead sea bream, focusing on skin and intestine microbiota and their associated shifts with growth and metabolic homeostatic markers in a crowding stress experiment of limited oxygen (O_2_) availability and temperature changes that mimicked the crowding and oxygen conditions of most Mediterranean farms during the summer on-growing finishing phase. To do this, a six-week trial during the summer was carried out to characterize the microbiome of two-year-old fish growing at three different stocking densities (LD, 6–8.5 kg/m^3^; MD, 12–17 kg/m^3^; HD, 22–25 kg/m^3^) under controlled water O_2_ concentrations (LD, 95–70% saturation; MD, 75–65% saturation; HD, 60–45% saturation). This study is part of an integrative behavioral, microbial, and transcriptional approach with the double aim of improving welfare monitoring and underscoring a series of stressful responses that serve to alert and adapt the organism in different ways.

## 2. Materials and Methods

### 2.1. Ethics Statement

All procedures were approved by the Ethics and Animal Welfare Committee of IATS and CSIC (2021/VSC/PEA/0192). Fish manipulation and tissue collection were carried out in a registered installation facility (ES120330001055) under the principles published in the European Animal Directive (2010/63/EU) and Spanish laws (Royal Decree RD53/2013) for the protection of animals used in scientific experiments.

### 2.2. Experimental Setup and Sampling

Fish used in the present study were sourced from the work of Holhorea et al. [26], carried out in a flow-through system following the natural changes in day length and temperature. Briefly, 462 two-year-old fish with an average body weight across experimental groups of 479.62 ± 3.43 g were pit-tagged and redistributed in duplicated 3000 L cylindrical tanks at three different stocking densities (LD, 6 kg/m^3^; MD, 12 kg/m^3^; HD, 22 kg/m^3^). Fish were then grown up from May to July (6 weeks) until they achieved rearing densities of 8.5 kg/m^3^ (LD), 17 kg/m^3^ (MD), and 25 kg/m^3^ (HD), with individually averaged specific growth rates of 0.66 ± 0.01, 0.62 ± 0.01, and 0.39 ± 0.01, respectively. Fish were fed daily (12:00 A.M.) with automatic feeders to near-visual satiety with a commercial diet (Biomar, Palencia, Spain). Water inlet and aeration were regulated daily to maintain differentially controlled water O_2_ concentration (LD: 5–6 ppm, 70–95% saturation; MD: 4–5 ppm, 55–75% saturation; HD: 3–4 ppm, 45–60% saturation). Salinity (38–40 ppt) and pH (8.1–8.2) were constant during all the experiments, and the water temperature increased from 19 °C to 26–27 °C according to seawater natural conditions. Weekly determinations of unionized ammonia were always below the toxic threshold level (<0.05 mg/L).

At the end of the trial, 12 fish per treatment (non-feeding fish) were individually monitored during two consecutive days with high-frequency recording data loggers (AEFishBIT) [27] attached to the operculum for the precise tracking of endogenous swimming activity and respiratory frequency rhythms in the rearing tanks with minimal animal disturbance [10,28]. The same fish were used for the assessment of fish appearance (fin damage and skin lesions), blood stress markers (glucose and cortisol), and liver and muscle gene expression signatures by customized PCR arrays of stress-responsive genes already reported in Holhorea et al. [26]. Additionally, as part of the specific analyses of the present study, different tissue samples were taken to analyze the mucosal microbiota and gut transcriptome. Briefly, skin mucus was collected by gently scraping the left side of the fish with a clean microscope slide from the operculum to the tail, avoiding the collection of blood, urine, and feces along with mucus. The collected skin mucus was stored in sterile tubes and immediately frozen at −80 °C until use. Concerning the anterior intestine (AI), tissue portions of approximately 0.4 cm were put into RNA later for gene expression analysis by RNA-seq. For microbial analysis, the remaining part of the AI was cut out, opened, washed with sterile PBS to remove non-adherent bacteria, and the mucus was scrapped off using the blunt edge of a sterile scalpel. The collected intestinal mucus was kept on ice in sterile tubes, and bacterial DNA was immediately extracted after the completion of sampling. 

### 2.3. Nucleic Acid Extraction

DNA from up to 200 μL of skin and intestinal mucus samples was extracted using a High Pure PCR Template Preparation Kit (Roche, Basel, Switzerland), including a lysozyme lysis step for optimized DNA extraction [46]. RNA from AI samples was extracted and processed for gene expression analyses as described elsewhere [46]. 

### 2.4. Nanopore 16S rRNA Gene Sequencing and Bioinformatic Analysis

For the skin mucus samples, the complete 16S rRNA gene (V1–V9) was sequenced using the ONT MinION (Oxford Nanopore Technologies, Oxford, UK) device and the 16S Barcoding Kit 1–24 (SQK-16S024), according to the manufacturer’s protocol, including modifications of input DNA and PCR conditions described elsewhere [47]. The amplified DNA was quantified using PicoGreen™ (Life Technologies, Carlsbad, CA, USA), and libraries of 100 fmol were loaded into the ONT MinION device. Libraries were sequenced using an R9.4/FLO-MIN106 flow cell and demultiplexed using MinKNOW v21.11.17. The sequencing was stopped when approximately an average of 100,000 reads per sample was achieved, which constituted a sequencing run time of 21–23 h. Between runs, the ONT-MinION flow cell was washed according to the ONT Flow Cell Wash Kit (EXP-WSH004) instructions.

After sequencing, basecalling was performed with Guppy v5.1.12, using the default parameters. The resulting FASTQ reads were pre-processed using Porechop v0.2.4 (https://github.com/rrwick/Porechop; accessed on 25 September 2022) for removing sequencing adapters from reads, NanoFilt v2.8.0 [48] for filtering reads below 1200 base pairs (bp) and above 1800 bp, and Yacrd v0.6.2 [49] for chimera detection and removal. Sequences were assigned as distinct amplicon sequence variants (ASVs) and subsequently mapped for taxonomy assignment with Minimap2 v2.17-r941 [50], using SILVA v138.1 [51] as the reference database. Raw sequence data were uploaded to the Sequence Read Archive (SRA) under Bioproject accession number PRJNA1039578 (BioSample accession numbers: SAMN38222399-429).

### 2.5. Illumina 16S rRNA Gene Sequencing of Gut Mucus Samples and Bioinformatics Analysis

For intestinal microbiota analysis, the V3-V4 region of the 16S rRNA gene was sequenced using the Illumina (San Diego, CA, USA) MiSeq platform (2 × 300 paired-end runs) at the Genomics Unit of the Madrid Science Park Foundation (FPCM), as described elsewhere [46]. Three samples, one per treatment, failed amplification and were removed from further analysis. FASTQ forward and reverse reads were quality-filtered and pre-processed using FastQC and Prinseq v0.20.4 [52]. Terminal N bases in both ends were trimmed, and sequences with >5% N bases, <150 bp long, a Phred quality score < 28 in both ends, or a Phred average score < 26 were discarded. Clean forward and reverse reads were merged with fastq-join [53]. For bacterial taxonomic assignment, reads were aligned using the VSEARCH database v2.15.1 and the BLAST database v2.8.1 [54,55]. Raw sequence data were uploaded to the Sequence Read Archive (SRA) under Bioproject accession number PRJNA1039578 (BioSample accession numbers: SAMN38222430-456).

### 2.6. Host Intestinal RNA Sequencing and Bioinformatic Analysis

Concerning the host gut transcriptomic analysis, 30 (10 fish/group) RNA-seq libraries were prepared and sequenced on an Illumina Novaseq 6000 platform in a 2 × 150 nucleotide paired-end (PE) read format according to the manufacturer’s protocol at the GENEWIZ company (Leiden, Germany). Details of the bioinformatic analyses are described elsewhere [36]. Briefly, quality analysis was performed with FastQC; libraries were filtered with Trimmomatic v0.40 [56] and mapped and annotated with Hisat2 v2.0.5 [57], using the CSIC gilthead sea bream genome as a reference [58]. Unique transcript hit counts were calculated by using featureCounts v1.5.0-p3 from the Subread package [59]. Raw sequence data were uploaded to the Sequence Read Archive (SRA) under Bioproject accession number PRJNA1039578 (BioSample accession numbers: SAMN38222457-486).

### 2.7. Statistics and Visualizations

For 16S rRNA gene sequencing data, rarefaction curves were obtained using the R package *phyloseq v1.41.1* [60]. For all analyses, sample depths were normalized by total sum scaling and made proportional to the total sequencing depth [61]. Differences in richness (Chao1 and ACE), diversity indexes (Shannon and Simpson), and phylum and microbial abundance were determined by the Kruskal-Wallis test followed by Dunn’s post-test, with a significance threshold of *p* < 0.05. The beta diversity across groups was tested with permutation multivariate analysis of variance (PERMANOVA) using the non-parametric method *adonis* (10,000 random permutations) in the R package *vegan* [62]. To study the separation between experimental groups, partial least-squares discriminant analyses (PLS-DA) were performed using EZinfo v.3.0 (Umetrics, Umeå, Sweden), and Hoteling’s T^2^ statistic was calculated using the same software to detect and report outliers in the model.

The contribution of the different taxonomies to the group separation was determined by the minimum variable importance in the projection (VIP) values, where a VIP score ≥ 1 was considered to be an adequate threshold to determine discriminant taxa in the PLS-DA model [63]. The quality of the PLS-DA model was evaluated by the parameters R2Y (cum) and Q2 (cum), which indicate fit and prediction ability, respectively. The Bioconductor R package *ropls* [64] were used to assess whether the supervised model was being overfitted (500 random permutations validation test). To determine the bacteria genera that most likely explain differences between the three experimental groups, a linear discriminant analysis (LDA) effect size (LEfSe) [65] was conducted using the Bioconductor R package *microbiomeMarker* [66] and bacteria taxa with VIP ≥ 1. 

For RNA-seq analyses, 6150 differentially expressed (DE) transcripts (*p* < 0.05, One-Way ANOVA) in at least one of the group comparisons were retrieved using DESeq2 [67]. These transcripts were used to construct a PLS-DA model, and the discriminant transcripts (VIP ≥ 1) were used to perform K-means analysis using iDEP.951 (http://bioinformatics.sdstate.edu/idep95/; accessed on 3 October 2022) to separate transcripts by expression patterns. Transcripts in the different clusters were analyzed by Fisher test-based over-representation analyses of gene ontology-biological process (GO-BP) terms using ShinyGO v 0.76 [68], and statistical significance was accepted at FDR < 0.05. Enriched GO-BP terms were clustered in arbitrary supra-categories, their relationships according to their shared transcripts were retrieved using the runGSA function of the *piano* R package [69], and the resulting networks were visualized with Cytoscape v3.8.2 [70].

For correlation analyses of changing bacteria from skin microbiota and gathered biomarkers, pairwise Spearman correlation coefficients were calculated for samples from the population of a given experimental group (HD fish). The corresponding *p*-values were calculated using the cor.test function of the *corrplot* R package [71]. Significant correlations at *p* < 0.01 and *p* < 0.05 were visualized with Cytoscape v3.8.2 [70].

## 3. Results

### 3.1. Skin Mucus Composition and Diversity Analysis

**Figure 1:**
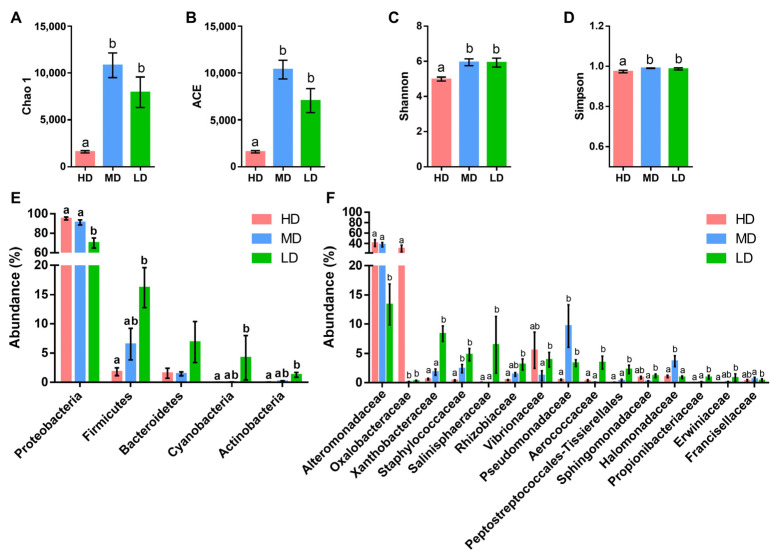
Skin microbiota, diversity, and composition. Species richness estimates (Chao 1 (**A**) and ACE (**B**)) and diversity indexes (Shannon (**C**) and Simpson (**D**)) of the bacterial communities from the skin of the gilthead sea bream reared at high (HD, n = 10), medium (MD, n = 10), and low (LD, n = 10) density; (**E**) bar chart representing the relative abundance in percentage of bacterial phyla in the different groups. Only phyla with an abundance higher than 0.5% in at least one group are shown; (**F**) bar chart represents the relative abundance in percentage of bacterial families in the different groups. Only families with an abundance higher than 0.5% in at least one group that show significant differences among groups are shown. Different letters represent statistical differences among groups within the same parameter or taxa (Kruskal–Wallis + Dunn’s, *p* < 0.05).

Results of the assessment of fish appearance, blood stress markers, and liver and muscle gene expression signatures can be found in Holhorea et al. [27]. The ONT-MinION sequencing yielded 3.2 million high-quality reads (107,600 mean reads per sample) that were taxonomically assigned at a mean rate of 92.2% (Table S1A). Rarefaction curves approximated saturation and showed good coverage of the bacteria community (Figure S1), allocated in five phyla and 25 families with ≥0.5% average abundance in at least one experimental group. Richness (Chao 1 and ACE) and alpha diversity (Shannon and Simpson) indexes were significantly lower in HD fish in comparison to MD and LD fish (Figure 1A–D). In all fish groups, the dominant phylum was Proteobacteria, with abundances varying from 90% (HD and MD fish) to 70% (LD fish). The second most abundant phylum was Firmicutes, which gradually increased from 2% to 15% with the decrease in stocking density. The same trend was followed by Cyanobacteria, Actinobacteria, and Bacteroidetes, though no significant changes were found in the case of Bacteroidetes (Figure 1E). At a family level (Figure 1F), the phylum Proteobacteria was mostly represented by Alteromonadaceae, with abundances varying from 40–37% in HD and LD fish to 12% in LD fish. Oxalobacteraceae was the second family most represented in HD fish (~40%), but its abundance decreased drastically in MD and LD fish, with abundances lower than 0.5%. Conversely, Pseudomonadaceae and Halomonadaceae (13% of the total abundance) were consistently more abundant in the MD group, whereas the highest abundance of Xanthobacteraceae, Staphylococcaceae, Salinisphaeraceae, Rhizobiaceae, and Aerococcaceae (26% of the total abundance) was achieved in LD fish.

**Figure 2:**
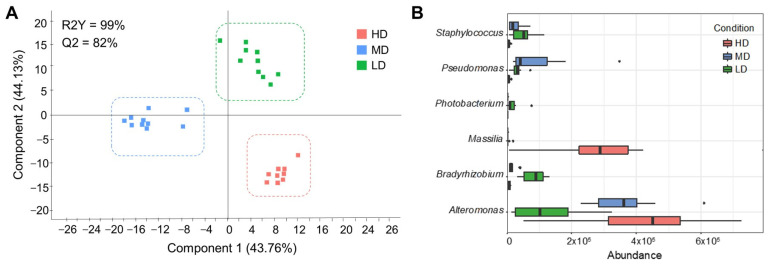
Skin microbiota, discriminant analyses, and biomarkers (**A**) Two-dimensional partial least squares discriminant analysis (*p* < 0.05) scores plot (PLS-DA) constructed using the taxonomic composition of the skin microbiota of gilthead sea bream reared at high (HD, n = 10, red dots), medium (MD, n = 10, blue dots), and low (LD, n = 10, green dots) density. Each square represents the distribution of the individual samples between the first two components in the model. (**B**) Linear discriminant analysis effect size analysis performed at the level of genus represents the significant biomarkers for each group and their abundance in normalized counts.

The number of skin mucus bacteria that are unique to each experimental group was regulated by stocking density, as shown in the PERMANOVA beta-diversity test (F = 7.405, R^2^ = 0.35, *p* < 0.001). Such microbiota differentiation was also evidenced by discriminant analysis that separated the three experimental groups with a correct classification of all individuals in each group (Figure 2A). The fitted PLS-DA model was statistically validated (R2Y (cum) = 99%; *p* < 0.05; Q2 (cum) = 82%; *p* < 0.05), explaining the two first components, 43.76% and 44.13% of the total variance. The fit of the PLS-DA model was validated by a permutation test (Figure S2). The resulting bacteria with significant VIP values (≥1) were 284 (Table S2A), which comprised almost the totality of the skin mucus bacteria taxa. After LEfSe analysis, the bacterial taxa with discriminant value were reduced to six genera, five of them belonging to Proteobacteria (*Alteromonas*, *Massilia*, *Pseudomonas*, *Bradyrhizobium*, and *Photobacterium*) and one to the Firmicutes (*Staphylococcus*) phyla (Figure 2B). At a closer look, a higher abundance of *Alteromonas* and *Massilia* was observed to be present in HD fish, while *Pseudomonas* was highly represented in MD fish. Conversely, *Staphylococcus*, *Bradyrhizobium*, and *Photobacterium* were overrepresented in LD fish.

### 3.2. Skin Mucus Correlation Network

**Figure 3:**
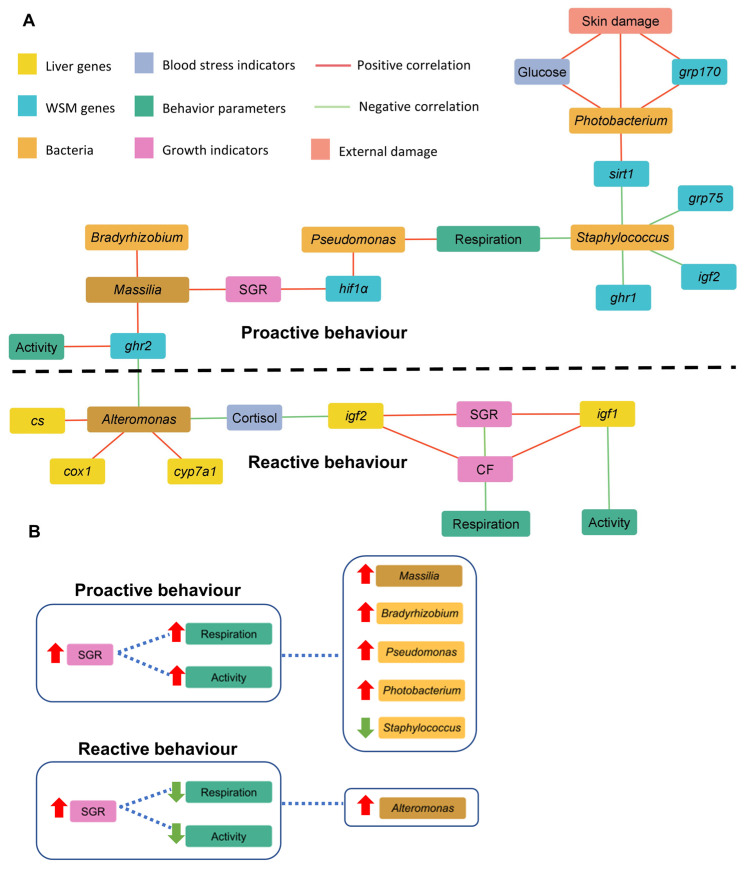
Skin microbiota and gathered biomarker analyses: (**A**) Correlation network performed on data from high-density reared fish (HD) showing significant positive (red lines) and negative (green lines) correlations (Spearman, *p* < 0.05) of discriminant skin microbiota (light orange, reduced abundance in HD; dark orange, increased abundance in HD) with biomarkers of external damage (red), growth (pink), behavior (green), blood stress indicators (purple), and liver (yellow) and muscle (blue) differentially regulated genes; (**B**) Correlation of *Massilia* with the regulation of biological processes at the local level indirectly correlated to proactive behavior. Its changes in abundance were related to other bacterial changes. Conversely, *Alteromonas* was related to depressed activity and respiratory rates, indirectly correlated to reactive behavior. *grp170*: Glucose-regulated protein 170 kDa; *sirt1*: Sirtuin 1; *grp75*: Glucose-regulated protein 75 kDa; *ghr1*: Growth hormone receptor 1; *hif1α*: Hypoxia inducible factor 1α; *ghr2*: Growth hormone receptor 2; *cs*: Citrate synthase; *cox1*: Cytochrome c oxidase subunit 1; *cyp7a1*: Cholesterol 7-Alpha-monooxygenase; *igf2*: Insulin growth factor 2; *igf1*: Insulin growth factor 1; SGR: Specific Growth Rate; CF: Condition Factor.

The results of the assessment of fish appearance, blood stress markers, and liver and muscle gene expression are described in Holhorea et al. [27]. Despite the over-representation of *Alteromonas* and *Massilia* in the skin mucus of HD fish, correlation network analysis evidenced an opposite trend for these two bacteria with the increase in stocking density (Figure 3A). An increased abundance of *Alteromonas* in HD fish was concurrent with an enhanced hepatic expression of growth (*igf1*, *igf2*), lipid metabolism (*cyp7a1*), and oxidative metabolism-related stress markers (*cs*, *cox1*). Conversely, a higher abundance of *Massilia* in the skin mucus of HD fish was concurrent with the up-regulated expression of seven genes related to growth (*ghr1*, *ghr2*, *igf2*), antioxidant defense (*grp170*, *grp75*), and energy metabolism (*sirt1*, *hif1α*), all of which (except *ghr1*) were up-regulated in HD fish in comparison to MD/LD fish. This integrative approach also rendered a different behavioral pattern, in which *Alteromonas* abundance and low plasma cortisol levels appeared related to depressed activity and respiratory rates (reactive behavior) that might contribute to preserving growth at high stocking densities through transcriptionally mediated changes at the hepatic (systemic) level. In addition, a higher abundance of *Massilia* was directly or ultimately correlated (*p* < 0.05) with the regulation of different biological processes at the local level (white skeletal muscle), which was concurrent with a proactive behavior (increased activity and respiratory rates) with increased signs of skin erosion due to the competition for available space and the distributed feed. Its changes in abundance were also related to other bacterial changes, which rendered an increased abundance of *Bradyrhizobium*, *Pseudomonas*, and *Photobacterium* in combination with a lower representation of *Staphylococcus* (Figure 3B). Data on bacterial taxa for correlation analysis can be found in Table S3B.

### 3.3. Intestinal Microbiota Composition and Diversity Analysis

**Figure 4:**
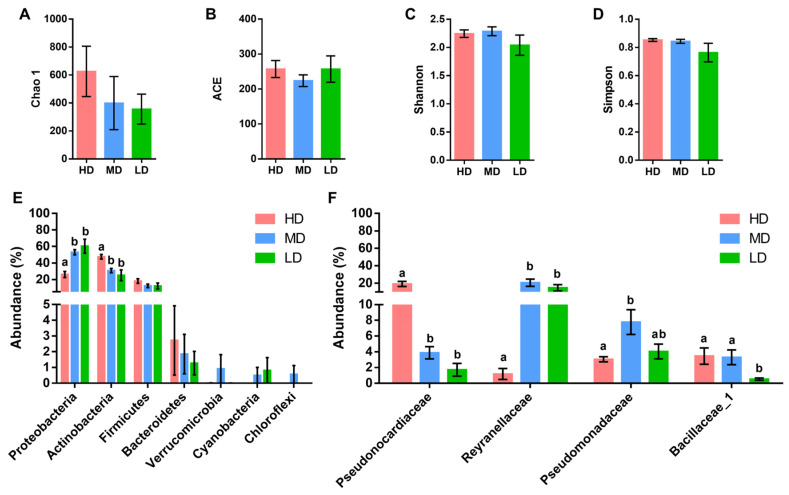
Gut adherent microbiota, diversity, and composition. Species richness estimates (Chao 1 (**A**) and ACE (**B**)) and diversity indexes (Shannon (**C**) and Simpson (**D**)) of the bacterial communities from the anterior intestine of gilthead sea bream reared at high (HD, n = 9), medium (MD, n = 9), and low (LD, n = 9) density; (**E**) bar chart representing the relative abundance in percentage of bacterial phyla in the different groups. Only phyla with an abundance higher than 0.5% in at least one group are shown; (**F**) bar chart represents the relative abundance in percentage of bacterial families in the different groups. Only families with an abundance higher than 0.5% in at least one group that show significant differences among groups are shown. Different letters represent statistical differences among groups within the same parameter or taxa (Kruskal–Wallis + Dunn’s, *p* < 0.05).

The Illumina MiSeq rendered 5.6 million raw reads (202,428 mean reads per sample) that were taxonomically assigned at a mean rate of 54% (Table S1B). Rarefaction curves approximated saturation and showed good coverage of the bacteria community (Figure S3), allocated in seven phyla and 23 families with more than 0.5% abundance in at least one experimental group. Richness estimates (Chao 1 and ACE) and alpha diversity (Shannon and Simpson) indexes were not significantly altered by the different stocking densities (Figure 4A–D). In all experimental groups, the dominant phyla were *Proteobacteria* (25–65%), *Actinobacteria* (21–48%), *Firmicutes* (9–18%), and *Bacteroidetes* (1.5–2%). *Proteobacteria* was the most abundant phylum in the MD and LD groups, while the *Actinobacteria* phylum was overrepresented in the HD group (Figure 4E). At the family level, the abundance of the *Pseudonocardiaceae* family (*Actinobacteria* phylum) increased to 20% with the highest stocking density, decreasing below 5% in the MD and LD groups. Likewise, the *Bacillaceae*_1 family belonging to the phylum *Firmicutes* significantly increased its abundance (7.8%) in comparison to MD/HD fish (3.9–3.0%). Conversely, the *Proteobacteria* family’s *Reyranellaceae* and *Pseudomonadaceae* (4.5% and 1.14% in total) were less abundant in HD fish than in MD/LD fish (24–12% and 7.4–3.6% in total) (Figure 4F). 

**Figure 5:**
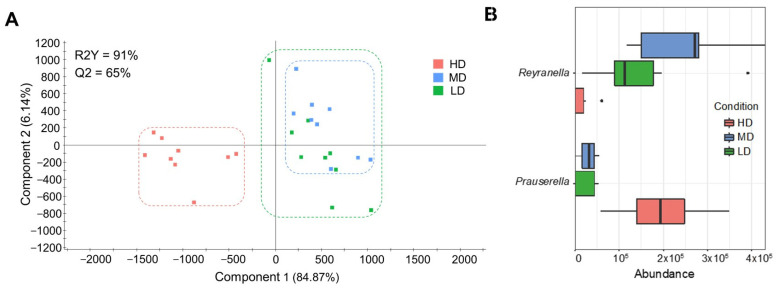
Gut adherent microbiota, discriminant analyses, and biomarkers: (**A**) Two-dimensional partial least squares discriminant analysis (*p* < 0.05) scores plot (PLS-DA) constructed using the taxonomic composition of the anterior intestine microbiota of gilthead sea bream reared at high (HD, n = 9, red dots), medium (MD, n = 9, blue dots), and low (LD, n = 9, green dots) density. Each square represents the distribution of the individual samples between the first two components in the model; (**B**) Linear discriminant analysis effect size analysis performed at the level of genus, representing the significant biomarkers for each group and their abundance in normalized counts.

Variations in intestinal microbiota composition with fish stocking were also evidenced by changes in beta-diversity (PERMANOVA beta-diversity test, F = 3.472, R^2^ = 0.22, *p* < 0.001). Such microbiota differentiation was reinforced by discriminant analysis, and the two first components of the fitted PLS-DA, putting together MD/LD fish, explained 91% of the observed variance (R2Y(cum), *p* < 0.05) and 65% of the predicted variance (Q2 (cum), *p* < 0.05) (Figure 5A). The fit of the PLS-DA model was validated by a permutation test (Figure S4). The number of bacteria taxa with significant VIP values (≥1) was 29 (Table S2B), representing more than 71% of the total bacteria population. LEfSe analysis identified the increased abundance of the *Prauserrella* genus (*Actinobacteria*) in concurrence with the decrease of *Reyranella* (*Proteobacteria*) as the most characteristic intestinal microbiota feature of our high density stocked fish (Figure 5B). 

### 3.4. Intestinal Wide-Transcriptomic Analysis

**Figure 6:**
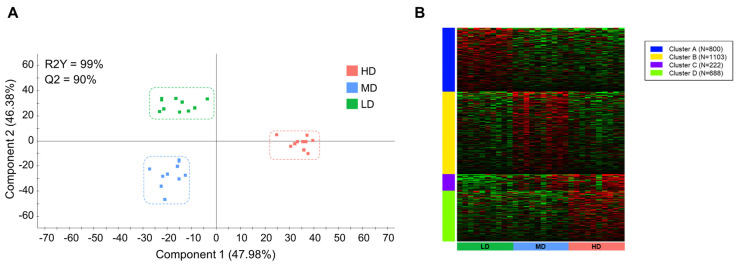
Gut transcriptome, discriminant, and K-means analyses: (**A**) Two-dimensional partial least squares discriminant analysis (*p* < 0.05) scores plot (PLS-DA) constructed using the expression values of all differentially expressed genes (DESeq2, *p* < 0.05) of the anterior intestine microbiota of gilthead sea bream reared at high (HD, n = 10, red dots), medium (MD, n = 10, blue dots), and low (LD, n = 10, green dots) density. Each square represents the distribution of the individual samples between the first two components in the model. (**B**) K-means analysis separating the 2813 discriminant genes (VIP > 1 in (A)) into four clusters based on the expression levels in the different groups (Z-score). Different colors indicate different clusters, with blue indicating cluster A (n = 800, higher expression in LD), yellow indicating cluster B (n = 1103, higher expression in MD), violet indicating cluster C (n = 222, higher expression in HD and intermediate in MD), and green indicating cluster D (n = 688, higher expression in HD).

A total of ~1823 million PE reads were obtained by RNA-seq, with an average of ~61 million reads per sample. After bioinformatic analysis (trimming, filtering, and mapping), ~90% of the total reads were mapped against the reference genome (Table S1C). A total of 6150 differentially expressed (DE) transcripts (5368 unique descriptions, UD) were retrieved by DESeq2 analysis, and the subsequent discriminant analysis separated the three experimental groups with a correct classification of all individuals in each group (Figure 6A). The resulting PLS-DA model was statistically validated (R2Y(cum) = 99%, *p* < 0.05; Q2 (cum) = 90%, *p* < 0.05), explaining the two first components more than 47.38% and 46.38% of the total variance. The fit of the PLS-DA model was validated by a permutation test (Figure S5). This separation was driven by 2813 transcripts (2212 UD) with significant VIP values (≥1) (Table S2C), which disclosed four different expression patterns after K-means clustering: Cluster A, 800 transcripts (699 UD) with the highest expression in LD; cluster B, 1103 transcripts (997 UD) with the highest expression in MD; cluster C, 222 transcripts (210 UD) with a gradual increase in expression with the rise of the stocking density (LD < MD < HD); cluster D, 688 transcripts (594 UD) with the highest expression in HD (Figure 6B).

### 3.5. Intestinal Mucus Correlation Network

**Figure 7:**
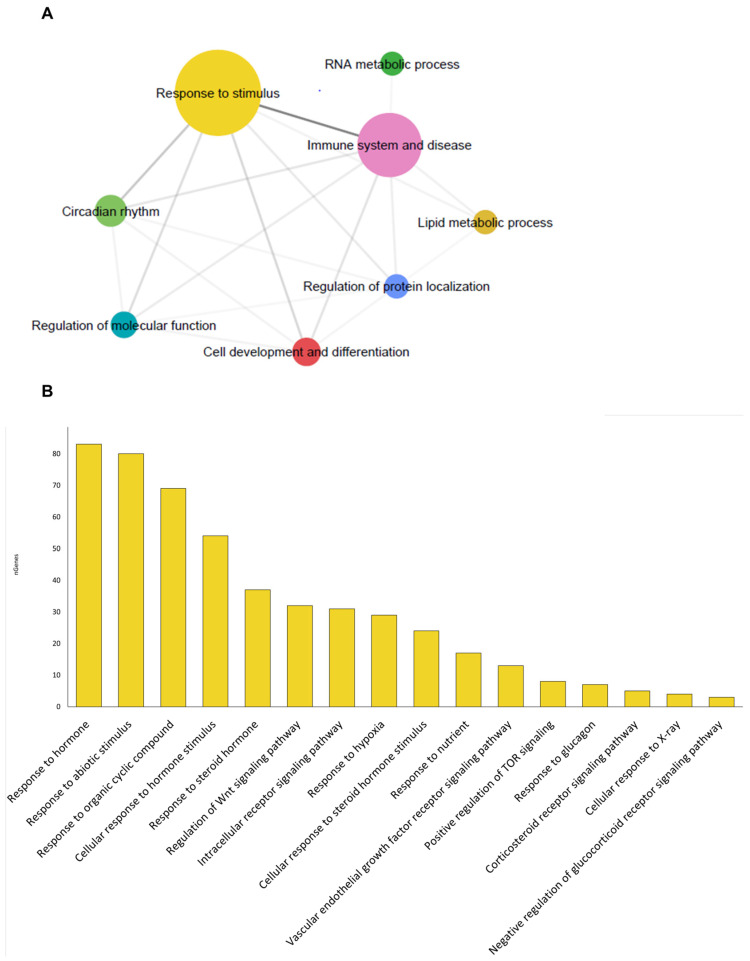
Gut transcriptome, gene ontology enrichment, and correlation with bacteria taxa: (**A**) Network layout representing the associations between the supra-categories of overrepresented GO-BP terms of gilthead sea bream according to their shared allocated terms. Node colors correspond to the representative name of the supra-category; (**B**) Bar chart showing the percentage of genes within the total identified in the “response to stimulus” supra-category that have been significantly correlated with the abundance of at least one bacterial taxa.

**Figure 8:**
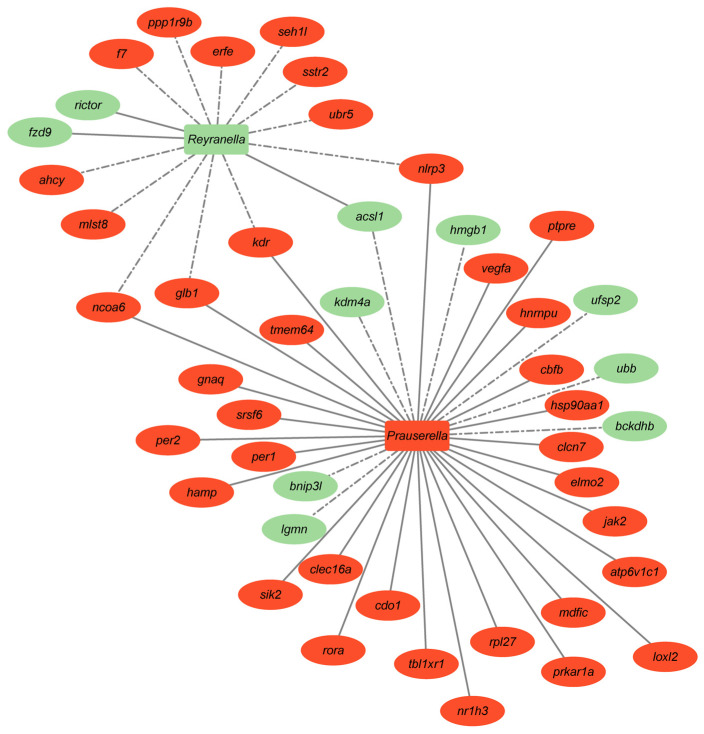
Host-bacteria Cross-talk. Correlation network showing significant positive (straight lines) and negative (dotted lines) correlations (Spearman, *p* < 0.01) between host differentially expressed genes (circles) contained in the response to stimulus-enriched supra-categories from Figure 4 and bacterial biomarkers (circles) from Figure 5B (VIP > 1, abundance >0.5% in at least one of the groups). *urbr5*: UBR5 ubiquitin protein ligase E3 component n-recognin 5; *sstr2*: Somatostatin receptor 2; *seh1l*: SEH1 Like Nucleoporin; *erfe*: Erythroferrone; *ppp1rgb*: Protein phosphatase 1 regulatory inhibitor subunit ubiquitin-protein1B; *f7*: Coagulation Factor VII; *ahcy*: Adenosylhomocysteinase; *mlst8*: Target of rapamycin complex subunit LST8; *rictor*: RPTOR Independent Companion Of MTOR Complex 2; *fzd9*: Frizzled Class Receptor 9; *acsl1*: Acyl-CoA synthetase long chain family member 1; *hmgb*: High Mobility Group Box 1; *ufsp2*: UFM1 Specific Peptidase 2; *ubb*: Ubiquitin B; *bckdhb*: branched chain keto acid dehydrogenase E1 subunit beta; *lgmm*: legumain; *bnip3l*: BCL2 Interacting Protein 3 Like; *kdm4*: Lysine Demethylase 4A; *ncoa6*: Nuclear Receptor Coactivator 6; *glb1*: Galactosidase Beta 1; *kdr*: Kinase Insert Domain Receptor; *nlrp3*: NLR Family Pyrin Domain Containing 3.

To understand the biological processes in which the DE transcripts within each cluster might be involved, an enrichment analysis was performed (Table S4). The enriched GO-BP terms were displayed and clustered in eight supra-categories: response to stimulus (189 transcripts), RNA metabolic process (8 transcripts), circadian rhythm (31 transcripts), immune system and disease (125 transcripts), lipid metabolic process (9 transcripts), regulation of molecular function (16 transcripts), cell development and differentiation (21 transcripts), and regulation of protein localization (9 transcripts) (Figure 7A). Focusing on the most abundant GO-BP supra-category, “Response to stimulus”, DE transcripts within 16 GO-BP terms were significantly correlated with at least one bacteria taxa of discriminant value (VIP ≥ 1) (Figure 7B). Filtering by *Reyranella* and *Prauserella*, Spearman correlations (*p* < 0.01) disclosed a complex correlation network where up to eight genes implicated in the response to hormones (*urbr5*, *sstr2*, *seh1l*, *erfe*, *ppp1rgb*, *f7*, *ahcy*, and *mlst8*) interacted in the network and were negatively correlated with *Reyranella*, whereas up to three genes (*rictor*, *fzd9*, *acsl1*) related to TOR signaling, Wnt signaling, and fatty acid metabolism were positively correlated. In contrast, seven genes (*hmgb*, *ufsp2*, *ubb*, *bckdhb*, *lgmm*, *bnip3l*, and *kdm4a*) mainly related to response to hormones, abiotic stimulus, and hypoxia were negatively correlated with *Prauserella*, while 29 genes mainly related among other processes to response to steroid hormone and organic cyclic compounds were positively correlated. Besides, *Reyranella* and *Prauserella* nodes were interconnected by five DE transcripts (*ncoa6*, *glb1*, *kdr*, *acsl1*, *nlrp3*), which served to interrelate an overall stimulatory rather than suppressive gut transcriptomic response with the increase in high stocking densities (Figure 8). Such a feature, triggered, in turn, an over-representation of DE genes belonging to K-means Cluster A (highly expressed genes in LD, 1506 UD) and Cluster C/D (highly expressed genes in HD, 2503 UD) in the gut microbiome-host transcriptome network. Of note, neither *Prauserella* nor *Reyranella* displayed significant correlations with liver or muscle DE expressed genes with the changing stocking density. Data on bacterial taxa for correlation analysis can be found in Table S3B.

## 4. Discussion

High stocking densities and limited O_2_ availability are prevalent aquaculture stressors with negative impacts on animal survival and productivity that become aggravated by higher temperatures [72]. Certainly, thermal stress increases the production of reactive oxygen species (ROS), and their negative effects in broilers and pigs are greater in fast-growing animals than in slow-growing animals with lowered mitochondrial and metabolic rates [73,74]. This improved thermo-tolerance with the decrease of basal metabolism is extensive to farmed fish, which makes the reduction of feed intake with high stocking densities, mild hypoxia, or thermal stress an adaptive response in nature [14,16]. Besides, the mitigating effects of a given drawback stressor serve to alleviate the negative impact of the other concurrent stressors. Hence, the impaired growth of gilthead sea bream in the range of 10–20 kg/m^3^ was avoided by maintaining the water O_2_ concentration above 55–70% saturation level [24,26,75]. In this way, the stocking density can be increased up to 36–44 kg/m^3^ without any evident drawback effect on gilthead sea bream growth performance when the water O_2_ concentration is maintained above 100% saturation [39], which is indicative of the complexity of the responses arising from crowding and hypoxia stress in fish [76]. This notion is supported at the transcriptional level by a tissue-specific orchestration of the stress response that reflects the different metabolic capabilities of each tissue as well as the nature and intensity of the hypoxic and crowding stress stimuli [14]. This is reinforced by the improvement of swimming performance by mild-hypoxia pre-conditioning through a muscle transcriptome reprogramming that persisted, at least in part, during a subsequent 3-week normoxia recovery period [77]. The association of high stocking density with changes in behavioral traits has also been established in gilthead sea bream, and it was noticeable that the perception of a higher competence for the available feed increased social cohesion among individuals [25,26]. Besides, the study of Holhorea et al. [26] displayed a growth-regulatory transition from systemic to local growth regulatory mechanisms, which might support proactive instead of reactive behavior. How these changes in behavioral traits can be driven or not by changes in the skin or gut microbiome is discussed below based on a host-16S rRNA-transcriptomics correlation network analysis. 

Experimental evidence in humans and animals shows that abnormal behavior is partly driven by changes in gut microbiota composition within the phylum Firmicutes, resulting in increased pro-inflammatory and lactic acid-producing bacteria and decreased butyrate-producing bacteria [78,79]. This gut dysbiosis is now recognized as a robust welfare marker, leading to efforts to establish a healthy core microbiota across organisms, particularly in farmed fish [80,81,82]. However, these efforts are challenged by the high variability of microbial composition within and among different populations. New approaches become necessary to overcome this variability and properly assess microbial dynamics [83]. The advent of next-generation sequencing (NGS) has revolutionized the study of complex microbial communities, with third-generation sequencing further advancing this field [84,85]. Third-generation sequencing enables cost-effective, real-time long-read sequencing, allowing for the use of the full 16S rRNA gene as a reliable phylogenetic marker [86,87]. Despite lower per-read quality accuracy (92–93%), long-read sequencing often results in lower taxonomic ambiguity compared to Illumina MiSeq V3-V4 amplified short-reads [88,89,90]. Optimized primer sets with the ONT MinION long-read sequencer have shown better resolution in discriminating human gut bacteria [91]. However, using the ONT commercial 16S Barcoding Kit can mask low-abundant but important taxa (e.g., Actinobacteriota and Bacteroidota) in gilthead sea bream gut microbiota compared to Illumina MiSeq results [47]. Therefore, our study used both Illumina MiSeq for intestinal microbiota and an in-house ONT sequencing system for mucosal skin microbiota.

Earlier studies in fish have demonstrated that skin mucus has evolved as a metabolically active tissue with important roles in respiration, ionic and osmotic regulation, excretion, locomotion, communication, sensory perception, thermal regulation, and immunological defense, among others [92,93]. Thus, in many species, including gilthead sea bream, it has been proven that several biochemical markers (e.g., cortisol, glucose, lactate, alkaline phosphatase, transaminases) of skin mucus changed significantly under acute and chronic stress [94,95]. Besides, different proteomic and multi-omics approaches integrating the skin tissue and mucus layer have identified several responsive markers reflecting the activation or inhibition of cell protein turnover and exudation machinery following overcrowding, hypoxia, and/or repeated exposure to a fast series of automated stressors [95,96,97,98]. Likewise, focusing on a microbial approach, Tapia-Paniagua et al. [99] highlighted that the presence of skin ulcers provides microenvironments that perturb both the mucus composition and microbial biodiversity, making farmed fish more vulnerable to diseases. There is also now evidence that repeated air exposure over 4 weeks alters the composition of the skin microbiota in gilthead sea bream [38], with an increased abundance of *Pseudoalteromonas*, *Rubritalea*, and other bacteria taxa from the *Actinobacteria* phylum. Conversely, in our crowding/hypoxia stress model, bacteria taxa from the *Actinobacteria* phylum were largely underrepresented in HD and MD fish, while *Proteobacteria*, followed by *Firmicutes* and *Bacteroidetes*, were largely the most abundant phyla in all the studied fish groups. Moreover, after LEfSe filtering, five out of the six most discriminant bacteria taxa belonged to the *Proteobacteria* phylum, making the increased abundance of *Alteromonas* and *Massilia* a characteristic feature of HD fish in our experimental model, whereas the other three *Proteobacteria* (*Staphylococcus*, *Bradyrhizobium*, and *Photobacterium*) were overrepresented in LD fish as a main distinctive feature. Despite this, comparisons with this and other skin microbiota studies are difficult, if not impossible, due to differences in sequencing platforms, fish strains, developmental stage, rearing system, nutritional condition, and nature and intensity of stress stimuli, among other biotic and abiotic sources of variation. In any case, from this and previous studies across farmed fish and wild fish populations inhabiting different geographical locations, it appears that the over-representation of *Proteobacteria* and *Bacteroidota* phyla is a main characteristic feature of the fish skin microbiota [35,82,100,101,102], though the relative proportion of each bacteria phylum can remain highly variable.

At a closer look, *Alteromonas* species are large bacteria that can degrade and utilize a broad spectrum of organic substrates. They can also produce and secrete a variety of extracellular enzymes contributing to the hydrolysis of biopolymers, including polysaccharides, proteins, nucleic acids, and lipids, which makes these bacteria members of the marine master recycler [103]. Likewise, *Massilia* is widely present in wild and farming aquatic environments [104,105,106], with species of this bacteria taxon showing an increased capacity for degrading high aromatic compounds, including polycyclic aromatic hydrocarbons (PAHs) [107]. Moreover, correlation analysis indicates that these two bacterial taxa were mutually exclusive in our HD fish despite their averaged over-representation at the high stocking density, which would be indicative that the changing *Alteromononas*/*Massilia* ratio represents different dynamic stages of skin microbiota competition and assembly. In that sense, integration of 16S rRNA sequencing with other multi-omics data on behavior, growth performance, and tissue-specific gene expression helped us in assessing the flow of information from one omics level to another, being indirectly correlated herein with the increased abundance of *Massilia* with proactive behavior and a transition towards muscle/locally regulated growth, while systemic growth regulation via the liver Gh/Igf system was related to a persistent reactive behavior that was coincident with a skin mucus predominance of *Alteromonas* over *Massilia* [26]. The varying contribution of systemic (via liver Gh/Igf axis) and local growth-promoting actions on global growth are indicative of a different welfare condition and metabolic readjustment of the endocrine-growth cascade through season, development, and in response to a broad range of stressor stimuli [77,108]. As stated before by Holhorea et al. [26], the way in which the growth-regulatory mechanisms are driven by a different threshold level of O_2_ sensors requires further warrant, though it is noteworthy that the expression of *hif1α*, a master regulator of hypoxia-mediated responses, was more sensitive to the changing crowding and hypoxic condition in muscle than in liver. Taken together, these findings also reveal potential bidirectional interactions between microbiota and behavioral responses, which would serve to provide a means of regulating an animal’s physiological state through adjusting interactions with the environment. However, caution should be taken when inferring a causal relationship in the absence of controlled trials that test the effects of probiotics and/or microbial transplants on behavioral and physiological responses [109]. In any case, animal welfare science must expand its scope and methodological approaches to encompass the investigation of positive welfare states alongside possible sources of suffering [110]. Only then will we be able to judge when and how we might intervene in wild and farmed animals’ lives in a reliable manner. 

From our results, it is also conclusive that the intestinal microbiota of gilthead sea bream was more resilient than the skin microbiota to crowding and hypoxia stimuli, which was consistent with the notion of a tissue-specific susceptibility of mucosal microbiota to a given environmental stressor. Thus, overall available data show that fish external mucosa frequently signal changes to temperature and diseases, whereas the gut microbiota is severely affected by antibiotic treatments and salinity [111]. This would also be the case in the present study, in which the magnitude of changes at the intestine level was less evident than those found on the skin. By contrast, previous studies have evidenced that the gut microbiota of gilthead sea bream is highly regulated by diet and host genetics [46,112,113]. Despite this, in the present study, it was noteworthy that the abundance ratio of *Reyranella*/*Prauserella* dramatically decreased with the increase in stocking density and limited O_2_ availability. Such a feature was the result of the opposite trend of *Reyranella* and *Prauserella*, which rendered a low and high abundance of these bacteria genera in HD fish, respectively. Importantly, the bacteria taxa of the genus *Reyranella* have been previously described as abundant and stable taxa in gilthead sea bream, regardless of genetic background [113]. Besides, *Reyranella* has been related to the production of phenazines, which are known to possess broad-spectrum antibiotic activity against diverse fungal, bacterial, and oomycete plant pathogens [114]. Less explored is the genus *Prauserella*, though its presence has been reported in a marine environment [115], and members of its taxonomic family (*Pseudonocardiaceae*) were related to the production of many nutritional factors and a broad range of secondary metabolites, including antibiotics, enzymes, and bioactive compounds [116]. In that sense, both *Reyranella* and *Prauserella* can be considered beneficial for the preservation of intestinal function and health in challenged gilthead sea bream, though it appears that their relative contribution to metabolic homeostasis is largely altered by the environment. 

The key role of intestinal health and function becomes reinforced by a transcriptional integrative approach, which highlights the relevance of the connection of *Reyranella* and *Prauserella* with a number of DE genes fitting to the response to stimulus-enriched supra-categories. Importantly, this host-gut microbiota system interaction drives a stimulatory rather than a suppressive transcriptional response that would involve four (*nlrp3*, *kdr*, *glb1*, *ncoa6*) out of five genes acting as interconnectors of the *Reyranella* and *Prauserella* nodes. The exception was the *acsl1* gene, a key lipid metabolism enzyme that catalyzes the conversion of long-chain fatty acids to their active form, acyl-CoAs; thus, it is suppressed expression in HD fish would serve to maintain monocytes and macrophages responsiveness following exposure to pro-inflammatory molecules produced after infection with gram-negative pathogens at a low threshold level [117]. The *nlrp3* inflammasome system is also involved in maintaining the stability of the gut’s immune system, and its enhanced expression in our HD fish would be viewed as an activated sensor that ultimately protects the body from damage and pathogen insults [118]. Conversely, both *kdr* and *ncoa6* act as main regulators of epithelial cell proliferation and differentiation [119,120], and their interconnection with the *Reyranella*/*Prauserella* system highlighted the contribution of gut microbiota in the regulation of mucosal cell turnover in environmentally challenged fish. This is also extended to other adaptive stress responses involving the up-regulated expression of *glb1*, which has been related to improved resistance to abiotic stressors [121,122].

## 5. Conclusions

The interconnection between fish microbiome and stress responsiveness is a growing area of research that is now considered vital to ensuring the development of sustainable and welfare-oriented aquaculture practices. In that sense, the results of the present study aimed to infer new laboratory and operational welfare indicators for increased stress resilience in the context of rising temperatures and intensive rearing conditions to cover the increasing demand for seafood-sustainable aquaculture products. It is noteworthy that high stocking densities, in conjunction with limited O_2_ availability, were associated with changes in both skin and intestinal mucosal microbial populations, though the skin appears especially responsive to environmental changes. In that sense, correlation networks allowed us to link skin microbial changes to a certain type of behavior and growth regulatory system, while the changes observed at the intestinal level would contribute to preserving intestinal function and integrity, maintaining highly regulated immune responses, and epithelial cell turnover, among other important physiological processes.

## Data Availability

All data can be found within the paper and the additional files. The sequencing data presented in this study is available at the Sequence Read Archive (SRA), Bioproject accession number PRJNA1039578 (16S rRNA gene sequencing ONT biosample accession numbers: SAMN38222399-429, Illumina biosample accession number: SAMN38222430-456, RNA sequencing biosample accession numbers: SAMN38222457-486).

## Supplementary Materials

The following supporting information can be downloaded at: https://www.mdpi.com/article/10.3390/microorganisms12071360/s1. Figure S1: Rarefaction curves obtained from the sequencing data of skin mucus samples; Figure S2: Validation (permutation test, 500 permutations) of the PLS-DA model for skin mucus samples; Figure S3: Rarefaction curves obtained from the sequencing data of the 27 intestinal mucosal samples; Figure S4: Validation (permutation test, 500 permutations) of the PLS-DA model for intestinal mucus samples; Figure S5. Validation (permutation test, 500 permutations) of the PLS-DA model for intestinal RNA-seq samples; Table S1: (A) Detailed sequencing data obtained in this study for skin mucosal samples, as well as the number and percentage of taxonomically assigned reads (B) Detailed sequencing data obtained in this study for intestinal mucosal samples, as well as the number and percentage of taxonomically assigned reads (C) Detailed sequencing data obtained in the RNA sequencing and analysis of intestinal samples; Table S2: (A) List of discriminant taxonomies of skin mucosal samples (VIP ≥ 1); (B) List of discriminant taxonomies of intestinal mucosal samples (VIP ≥ 1); (C) List of discriminant transcripts of intestinal mucosal samples (VIP ≥ 1); Table S3: (A) Normalized count values of skin biomarkers; (B) Normalized count values of gut biomarkers; Table S4: List of GO-BP terms resulting from the Shiny GO enrichment analysis.
