# Supplementary material for: Differential Reshaping of Skin and Intestinal Microbiota by Stocking Density and Oxygen Availability in Farmed Gilthead Sea Bream (Sparus aurata): A Behavioral and Network-Based Integrative Approach
Source: Microorganisms. 2024 Jul 2;12(7):1360. doi: 10.3390/microorganisms12071360 (PMC11278760; doi:10.3390/microorganisms12071360)

Figure S4. Validation (permutation test, 500 permutations) of the PLS-DA model for intestinal mucus samples.

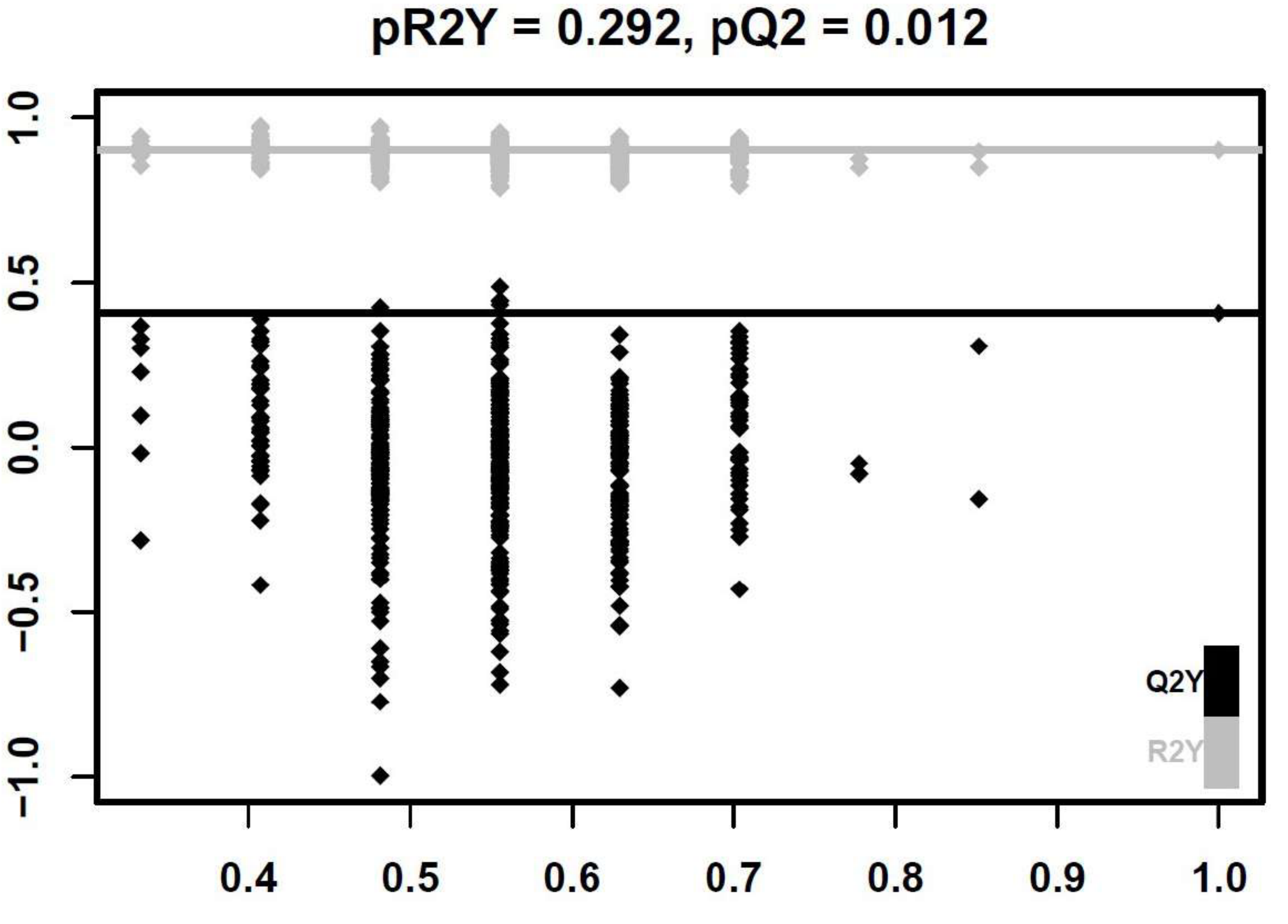

Supplement: Supplementary file 1 [file microorganisms-12-01360-s001.zip › Figure S4 Additional file 7.pdf]
