# Supplementary figures and images for: Differential Reshaping of Skin and Intestinal Microbiota by Stocking Density and Oxygen Availability in Farmed Gilthead Sea Bream (Sparus aurata): A Behavioral and Network-Based Integrative Approach
Source: Microorganisms. 2024 Jul 2;12(7):1360. doi: 10.3390/microorganisms12071360 (PMC11278760; doi:10.3390/microorganisms12071360)

**Figure S1.** Rarefaction curves obtained from the sequencing data of skin mucus samples.

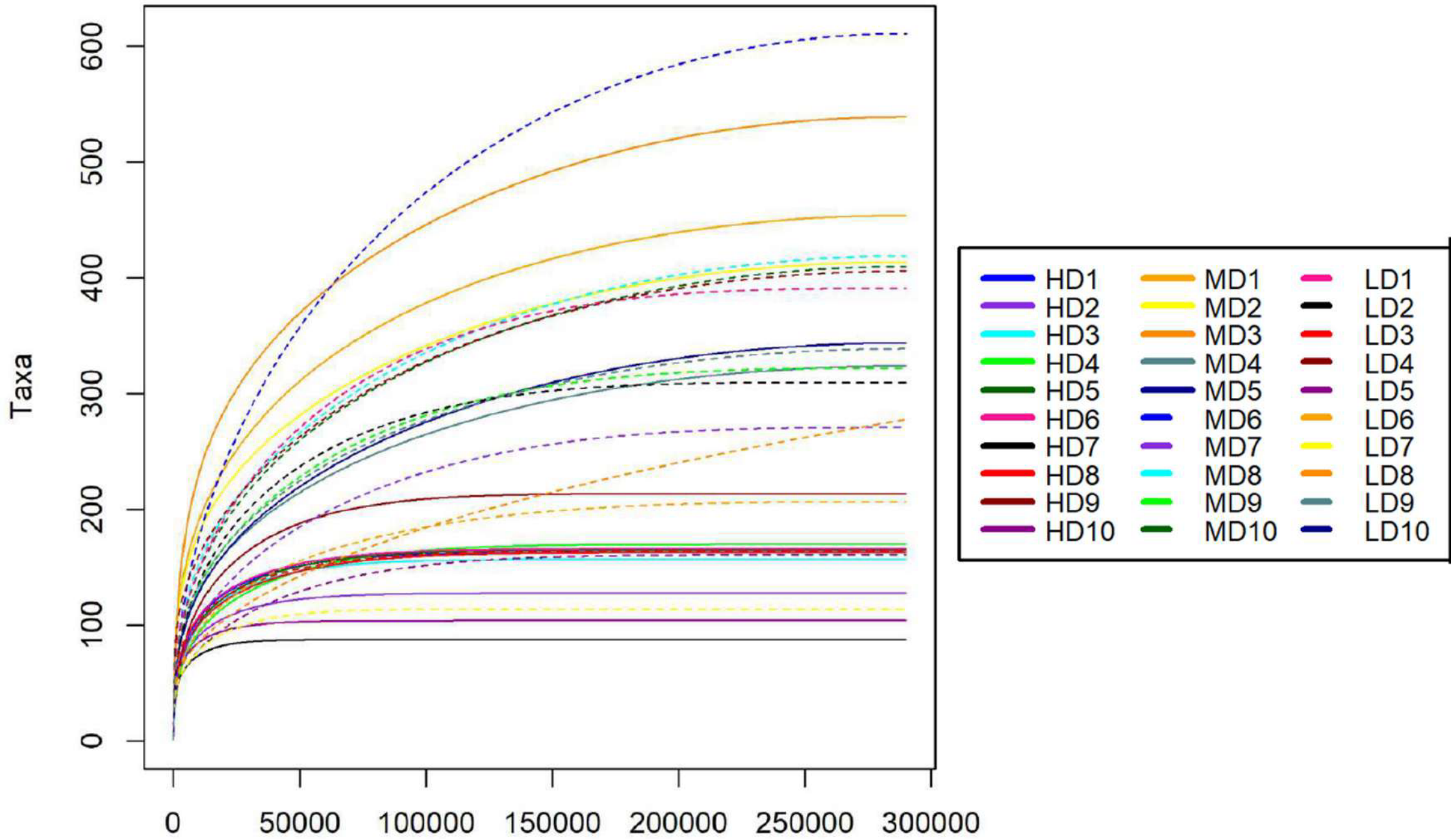

Supplement: Supplementary file 1 [file microorganisms-12-01360-s001.zip › Figure S1 Additional file 2.pdf]

**Figure S2.** Validation ( permutation test, 500 permutations) of the PLS-DA model for skin mucus samples.

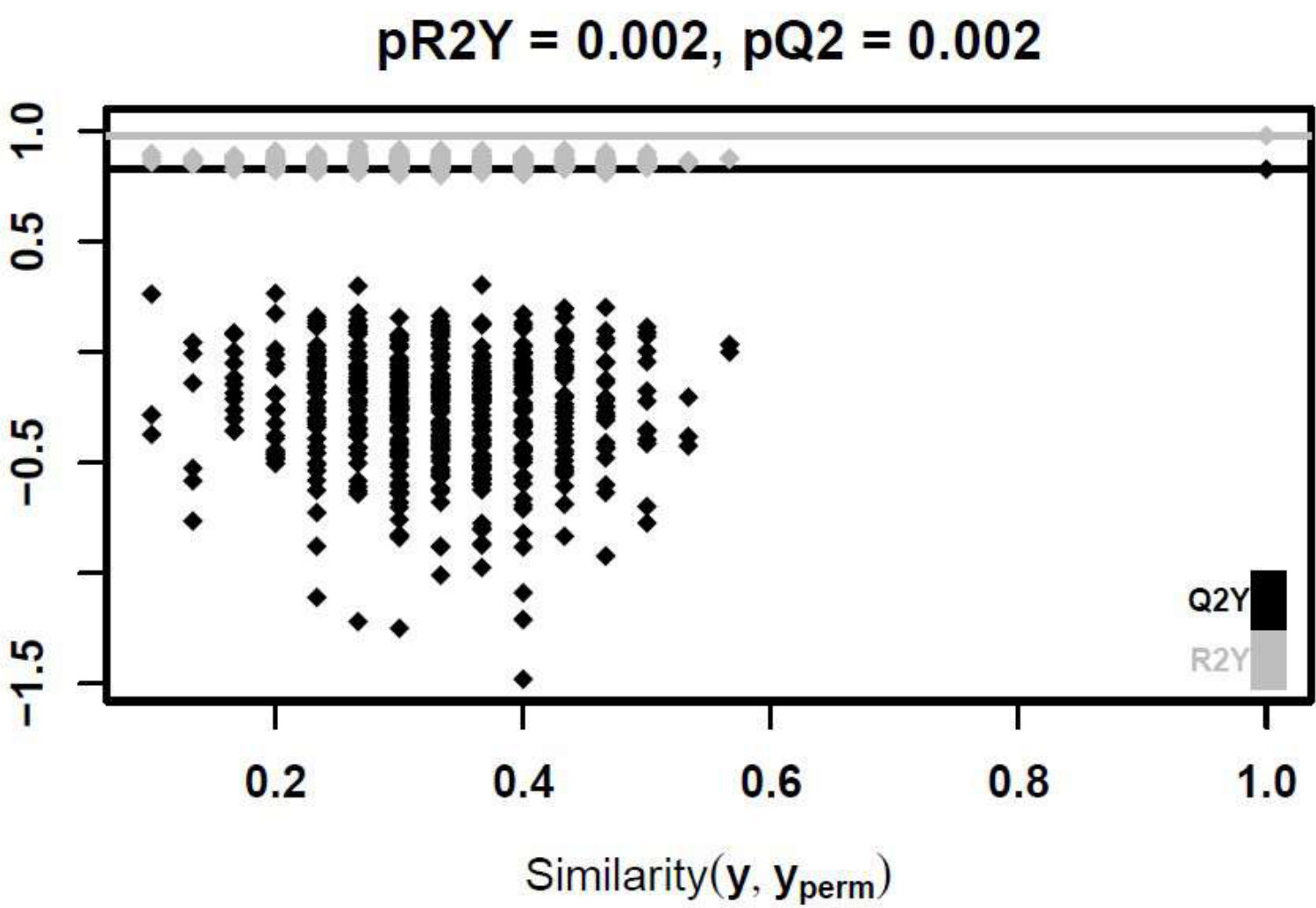

Supplement: Supplementary file 1 [file microorganisms-12-01360-s001.zip › Figure S2 Additional file 3.pdf]
